# Investigating an outbreak of measles in Kamwenge District, Uganda, July 2015

**DOI:** 10.11604/pamj.supp.2018.30.1.15269

**Published:** 2018-05-17

**Authors:** Alex Riolexus Ario, Fred Nsubuga, Lilian Bulage, Bao-Ping Zhu

**Affiliations:** 1 Uganda Public Health Fellowship Program, Kampala, Uganda; 2 Ministry of Health, Kampala, Uganda; 3 US Centers for Disease Control and Prevention, Uganda

**Keywords:** Outbreak investigation, measles, Uganda

## Abstract

Globalization has opened many fronts for disease outbreaks because of the quick movement of people and porous borders around the world. The emergence of zoonotic diseases and other communicable diseases highlights the need for implementation of the Global Health Security Agenda packages if countries are to achieve compliance with International Health Regulations (IHR 2005). Health workforce development is one of the critical components that must be addressed with utmost urgency if gaps in early disease detection and response are to be addressed. In this regard, this case study is based on a measles outbreak investigation in Uganda simulating a real-life outbreak investigation by field epidemiologists and seeks to demonstrate the principles of applied epidemiology outlining the critical steps in outbreak investigations and generation of evidence for decision making. It aims to shore up the health workforce capacity by providing practical training for field epidemiology students and professionals that builds their skills in outbreak investigation. This case study can be completed in less than three hours.

## How to use this Study

**General instructions:** the participants will read the paragraphs in the participant’s guide in turns as guided by the instructor. One instructor will facilitate the case study for a class of 10 – 15 students/residents. The instructor may ask for group discussion of an answer or direct a question to one particular participant. For questions that require calculations, the instructor will allow each participant to participate to maximize learning. The instructor may ask for a role play as necessary and also split the class into groups to generate brainstorming. The instructor’s guideprovides sufficient background materials to facilitate learning without review of further references.

**Audience:** residents in Intermediate and Advanced Field Epidemiology Training Programs.

**Prerequisites:** before using this case study, case study participants should have received lectures in defining outbreaks, conducting basic outbreak investigations, and basic biostatistics. Participants should have a basic science degree or equivalent knowledge of science.

**Materials needed:** flip chart or white board, markers, laptop with Microsoft Excel and Epi Info™ packages.

**Level of training and associated public health activity**: intermediate outbreak investigation.

**Time required**: 2-3 hours

**Language**: English

## Competing interest

The authors declare no competing interest.
